# Elimination of atrial fibrillation trigger from superior vena cava using a circular pulsed field ablation catheter

**DOI:** 10.1093/ehjcr/ytaf637

**Published:** 2025-12-11

**Authors:** Seigo Yamashita, Kosuke Minai, Michifumi Tokuda, Teiichi Yamane

**Affiliations:** Division of Cardiology, The Jikei University Katsushika Medical Center, 6-41-2 Aoto, Katsushika-ku, Tokyo 125-8506, Japan; Division of Cardiology, The Jikei University Katsushika Medical Center, 6-41-2 Aoto, Katsushika-ku, Tokyo 125-8506, Japan; Division of Cardiology, Department of Internal Medicine, The Jikei University School of Medicine, 3-19-18 Nishishinbashi, Minato-ku, Tokyo 105-8471, Japan; Division of Cardiology, Department of Internal Medicine, The Jikei University School of Medicine, 3-19-18 Nishishinbashi, Minato-ku, Tokyo 105-8471, Japan

## Summary

There was limited evidence regarding the efficacy and safety of pulsed field ablation (PFA) for the superior vena cava (SVC).^1,2^ We report the first case of incessant recurrent atrial fibrillation (IRAF) originating from the distal SVC, which could be successfully treated using circular PFA. Four PFA applications just below the firing site immediately eliminated IRAF, with high-resolution mapping confirming cessation of firing and SVC isolation at the PFA catheter level. Our approach enables us to accurately identify the PFA catheter position within the SVC and confirm good tissue contact with the SVC via venography. Together, the accurate prediction of the isolation line using bipolar pulses between circular electrodes may facilitate safe and effective SVC isolation.

## Case description

**Figure 1:**
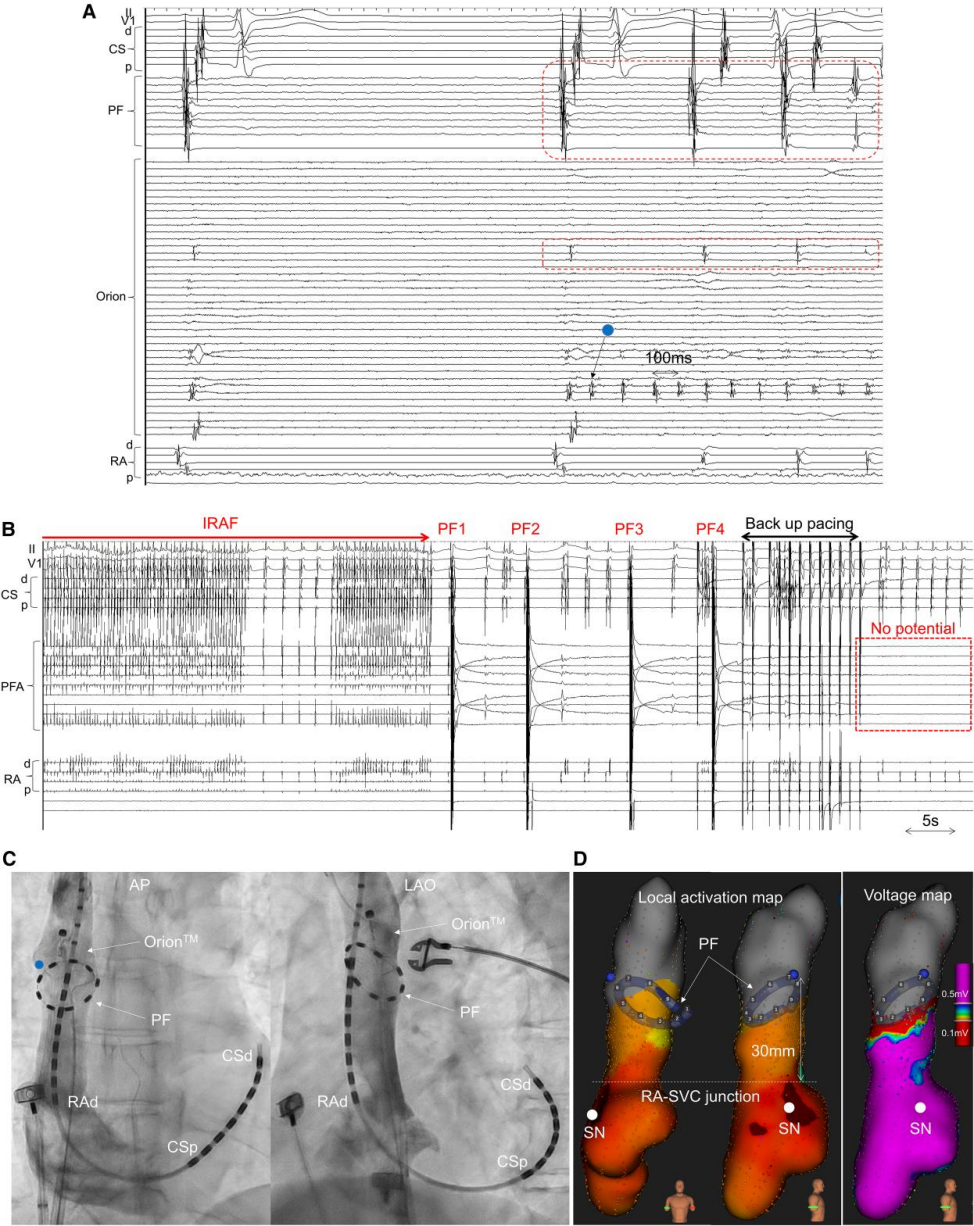
(*A*) Initiation of very rapid firings (cycle length: 100 ms) from the limited small area at the anterolateral location was detected at a spline of Orion^TM^ catheter (arrow). The other splines and pulsed field ablation catheter in the superior vena cava showed slow activity (dotted box). (*B*) Incessant recurrent atrial fibrillation disappeared just after four pulse trains, and the superior vena cava was simultaneously isolated (dotted box). (*C*) Venography demonstrated good contact to the superior vena cava in both anteroposterior (AP) and left anterior oblique (LAO) views. (*D*) Local activation map and voltage map during sinus rhythm after pulsed field ablation application showed successful superior vena cava isolation just above the circular pulsed field catheter position, with clear boundaries of the low voltage area. CS, coronary sinus; PF, pulsed field ablation catheter; RA, right atrium; SN, sinus node.

A 61-year-old man underwent catheter ablation for paroxysmal atrial fibrillation (AF) using a circular pulsed field ablation (PFA) catheter (PulseSelect^TM^, Medtronic, Minneapolis, MN, USA). Following the insertion of the PFA catheter into the left atrium, incessant recurrent AF (IRAF) was induced. The detailed mapping revealed the trigger from the superior vena cava (SVC), with very rapid myocardial firing (cycle length: 100 ms) at a localized area on the anterolateral aspect of the distal SVC. The activation spread outwards with conduction block (*Figure 1A*). After positioning the PFA catheter (array diameter: 25 mm) below the SVC firing site and confirming good contact by venography (*Figure 1C*), we delivered four biphasic, bipolar pulse trains. This application immediately eliminated IRAF and achieved SVC isolation (*Figure 1B*). The SVC was isolated at precisely at the same level where the PFA catheter was positioned, as evidenced by a well-demarcated low-voltage area boundary (*Figure 1D*). We thereafter successfully performed pulmonary vein isolation using PFA and completed the procedure after confirming the absence of PV/SVC reconnection and non-PV foci under administration of isoproterenol and adenosine. No sinus node or phrenic nerve injury was observed, and no atrial arrhythmia recurred during the 9-month follow-up.

## Data Availability

Data are available in a repository and can be accessed via a DOI link.

## References

[1] Pierucci N, La Fazia VM, Mohanty S, Schiavone M, Doty B, Gabrah K, et al Results of ICE-guided isolation of the superior vena cava with pulsed field ablation. JACC Clin Electrophysiol 2025;11:752–760. 39846925 10.1016/j.jacep.2024.11.009

[2] Ollitrault P, Chaumont C, Font J, Manninger M, Conti S, Matusik PT, et al Superior vena cava isolation using a pentaspline pulsed-field ablation catheter: feasibility and safety in patients undergoing atrial fibrillation catheter ablation. Europace 2024;26:euae160. 38875490 10.1093/europace/euae160 PMC11252500

